# Ferroptosis contributing to cardiomyocyte injury induced by silica nanoparticles via miR-125b-2-3p/HO-1 signaling

**DOI:** 10.1186/s12989-024-00579-5

**Published:** 2024-04-01

**Authors:** Xueyan Li, Hailin Xu, Xinying Zhao, Yan Li, Songqing Lv, Wei Zhou, Ji Wang, Zhiwei Sun, Yanbo Li, Caixia Guo

**Affiliations:** 1 Department of Occupational Health and Environmental Health, School of Public Health, Capital Medical University, No. 10 Xitoutiao, You An Men, Beijing, 100069 China; 2 Beijing Key Laboratory of Environmental Toxicology, Capital Medical University, No. 10 Xitoutiao, You An Men, Beijing, 100069 China; 3 Department of Toxicology and Sanitary Chemistry, School of Public Health, Capital Medical University, No. 10 Xitoutiao, You An Men, Beijing, 100069 China; 4 Department of Pharmaceutical Sciences, Beijing Institute of Radiation Medicine, Beijing, 100850 China; 5 Chaoyang District Center for Disease Control and Prevention, Beijing, 100021 China

**Keywords:** Silica nanoparticles, Myocardial injury, Ferroptosis, HO-1, miR-125b

## Abstract

**Background:**

Amorphous silica nanoparticles (SiNPs) have been gradually proven to threaten cardiac health, but pathogenesis has not been fully elucidated. Ferroptosis is a newly defined form of programmed cell death that is implicated in myocardial diseases. Nevertheless, its role in the adverse cardiac effects of SiNPs has not been described.

**Results:**

We first reported the induction of cardiomyocyte ferroptosis by SiNPs in both in vivo and in vitro*.* The sub-chronic exposure to SiNPs through intratracheal instillation aroused myocardial injury, characterized by significant inflammatory infiltration and collagen hyperplasia, accompanied by elevated CK-MB and cTnT activities in serum. Meanwhile, the activation of myocardial ferroptosis by SiNPs was certified by the extensive iron overload, declined FTH1 and FTL, and lipid peroxidation. The correlation analysis among detected indexes hinted ferroptosis was responsible for the SiNPs-aroused myocardial injury. Further, in vitro tests, SiNPs triggered iron overload and lipid peroxidation in cardiomyocytes. Concomitantly, altered expressions of TfR, DMT1, FTH1, and FTL indicated dysregulated iron metabolism of cardiomyocytes upon SiNP stimuli. Also, shrinking mitochondria with ridge fracture and ruptured outer membrane were noticed. To note, the ferroptosis inhibitor Ferrostatin-1 could effectively alleviate SiNPs-induced iron overload, lipid peroxidation, and myocardial cytotoxicity. More importantly, the mechanistic investigations revealed miR-125b-2-3p-targeted HO-1 as a key player in the induction of ferroptosis by SiNPs, probably through regulating the intracellular iron metabolism to mediate iron overload and ensuing lipid peroxidation.

**Conclusions:**

Our findings firstly underscored the fact that ferroptosis mediated by miR-125b-2-3p/HO-1 signaling was a contributor to SiNPs-induced myocardial injury, which could be of importance to elucidate the toxicity and provide new insights into the future safety applications of SiNPs-related nano products.

**Graphical Abstract:**

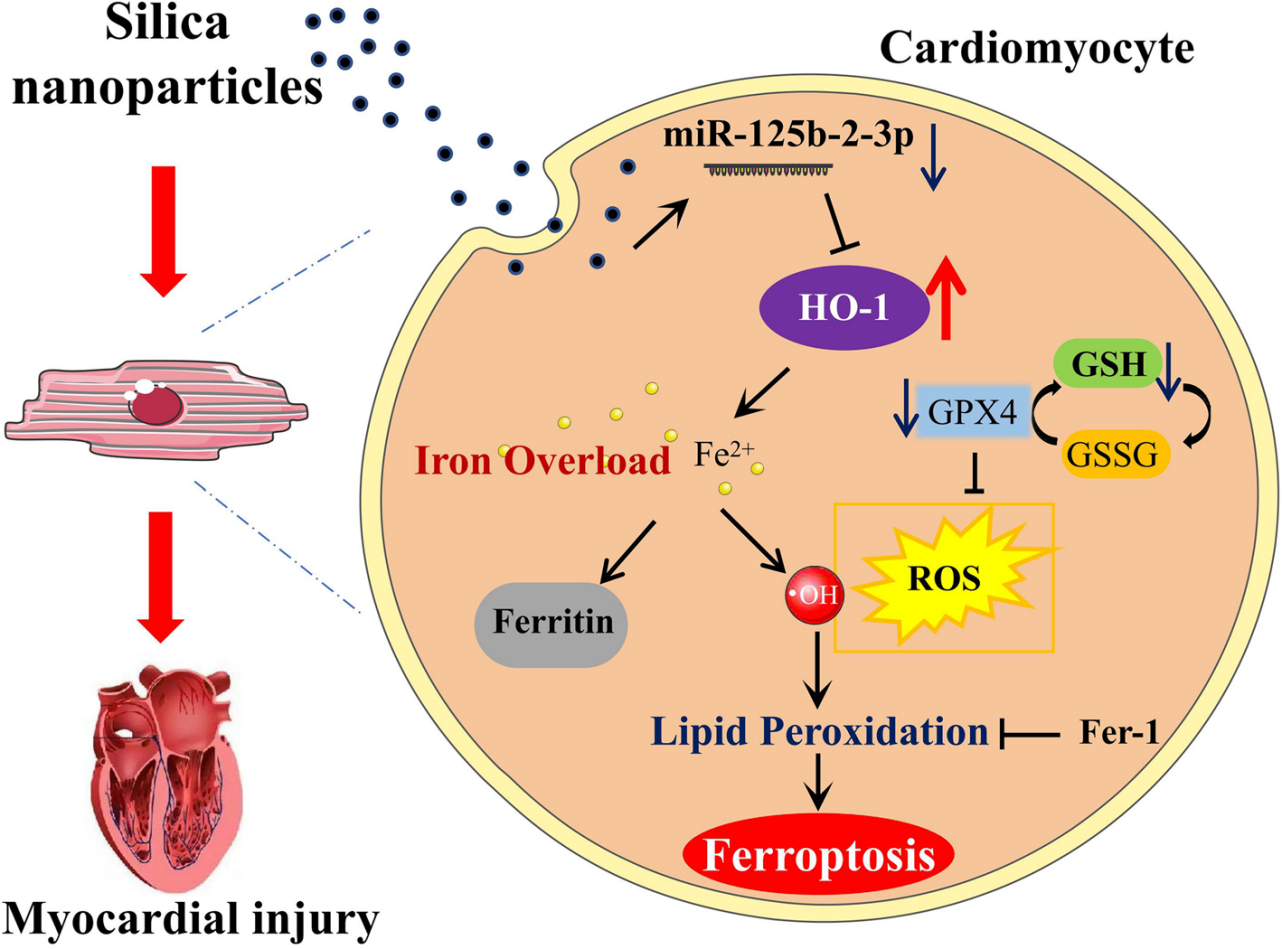

**Supplementary Information:**

The online version contains supplementary material available at 10.1186/s12989-024-00579-5.

## Introduction

With the development of nanotechnology, adverse effects of nanomaterials on human health have been a great concern due to their globally large-scale production and extensive applications. Amorphous silica nanoparticles (SiNPs) are one of the dominant nanomaterials in the global nano-market, with an output of up to 3.348 million tons in 2015 (https://www.grandviewresearch.com). Due to special properties, e.g., larger specific surface area, active centers, and easy synthesis, SiNPs have been widely applied in food industries, rubber, paint, paper manufacturing, and even biomedicine [44]. SiNPs have been used as a core drug carrier in the treatment of myocardial ischemia-reperfusion injury in rats [36]. As early as 2003, a paper in *Science* first proposed the concern about the potential toxicity of nanoparticles (NPs) [51]. However, a series of major issues on the safety evaluation of nanomaterials remain to be addressed.

Despite respiratory inhalation as a preliminary way for NP exposure to human beings, the systemic distribution of inhaled NPs hinted at their potential deleterious impacts on extrapulmonary organs or tissues [27]. A six-month follow-up study suggested nanomaterial handling was correlated with declined levels in antioxidant enzymes (GPx and SOD) and cardiovascular markers (vascular cell adhesion molecule, paraoxonase), and also lung function parameters [33]. Reportedly, silicon (Si) is known as a common component in airborne NPs [4]. Currently, accumulative epidemiological investigations have highlighted the contribution of airborne ultrafine particles (UFPs) to the increased incidence and mortality of cardiovascular diseases [2]. Besides, growing experimental evidence confirmed cardiovascular system acts as one of the most important targets upon NP exposure. For instance, Du et al*.* confirmed the blood and cardiac distribution of intratracheally instilled SiNPs, contributing to endothelial dysfunction and cardiac injury [12]. Consistently, SiNPs were detected in myocardial tissues in the ex-vivo heart perfusion model [39], leading to the impairment of cardiac relaxation. Feng et al*.* revealed the cardiac systolic dysfunction after intratracheal instillation of SiNPs in rats [15]. Liu et al*.* even reported the induction of tachyarrhythmias and lethal bradyarrhythmias in adult mice within 90 min after SiNP exposure via intravenous injection [37]. In the light of the literature [19], the cardiotoxicity elicited by SiNPs was ascribed to oxidative stress, inflammatory response, perturbations of ion channels, mitochondrial dysfunction, etc. Nevertheless, the detailed biological effects of SiNPs on heart tissue, especially under a long-term exposure mode, and its related molecular mechanisms are still utterly limited.

Ferroptosis is an iron-dependent programmed cell death that was proposed around 2012, with the characterizations of iron overload, glutathione peroxidase 4 (GPX4) decrease, reactive oxygen species (ROS) accumulation, and lipid peroxidation [9]. In terms of morphology, biochemistry, immune status, etc., ferroptosis is distinguished from several other forms of programmed cell death [22]. Currently, emerging studies highlighted the importance of ferroptosis in the pathogenesis of cardiovascular diseases, CVDs [14, 35]. Notwithstanding, the participation of ferroptosis in SiNPs-elicited cardiotoxicity remains unknown. Accumulated evidence confirms oxidative stress and lipid peroxidation responsible for the SiNPs-elicited myocardial death and ensuing cardiac dysfunction [39, 48]. A few recent reports reveal the participation of ferroptosis in SiNPs-elicited toxicity on vascular endothelial cells [25], microglia [24], and hepatocytes [32] in vitro. In this context, the in-depth investigations to reveal the role of ferroptosis in SiNPs-elicited myocardial toxicity, would facilitate a full understanding of the deleterious cardiac outcomes upon SiNP exposure, explore potential strategies for disease prevention and control, and provide new insight into the safety applications for nanoproducts.

Based on these issues, we aimed to investigate the participation of ferroptosis in SiNPs-induced cardiac injury using both in vivo and in vitro models, and to explore underlying mechanisms. Firstly, a sub-chronic exposure model of SiNPs was established in Wistar rats via intratracheal instillation. Apart from the evaluation of cardiac injury caused by SiNPs, the induction of ferroptosis was preliminarily assessed by detecting serum and cardiac iron levels, ROS, lipid peroxidation, and well-known ferroptosis regulators. Further, the induction of ferroptosis by SiNPs was also verified in in vitro cultured human myocardial cell line, AC16. Ferrostatin-1 (Fer-1), an inhibitor for ferroptosis, was applied to reveal the contribution of ferroptosis to cardiomyocyte damage caused by SiNPs. Lastly, the mechanistic investigations were performed, and our data suggested miR-125b-2-3p-targeted heme oxygenase-1 (HO-1) was responsible for SiNPs-induced cardiomyocyte ferroptosis through regulating intracellular iron metabolism to promote iron deposition and lipid peroxidation.

## Results

### SiNPs characterization

**Fig. 1:**
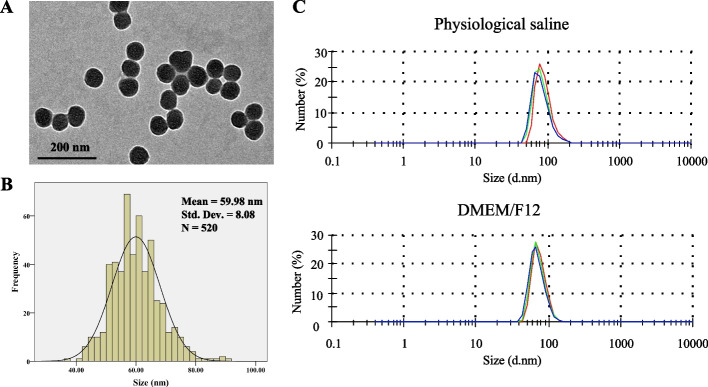
SiNPs characterization. **A** TEM image. Scale bar, 200 nm. **B** Particle size distribution. **C** Hydrodynamic size determination of SiNPs in dispersion media

Prior to the nanotoxicological evaluation, the particle characterization was carried out. SiNPs were approximately spherical and had good dispersion under the transmission electron microscope (TEM) observation (Fig. 1A), with an average particle size of 59.98 nm (Fig. 1B). Moreover, the hydrodynamic size and Zeta potential measurement data suggested the applied SiNPs maintained good stability and dispersibility in physiological saline and DMEM/F12 (Fig. 1C and Table S1).

### SiNPs induced myocardial injury in vivo

**Fig. 2:**
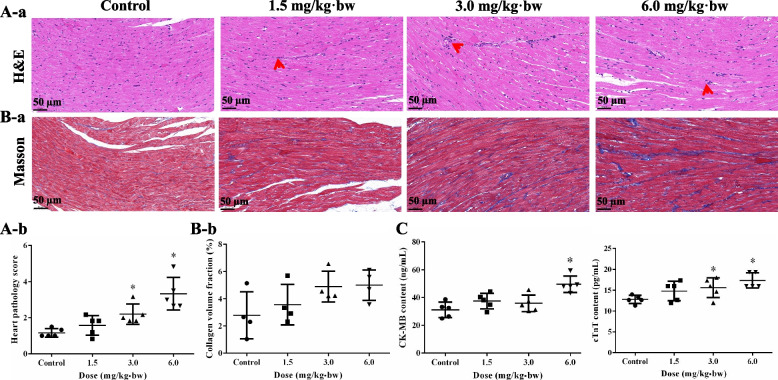
Myocardial injury in Wistar rats induced by SiNPs. **A** H&E staining of the heart tissues (**a**) and corresponding pathological score (**b**). Arrows: inflammatory cells. *n* = 5. Scale bar, 50 μm. **B** Masson staining (**a**) and collagen volume fraction analysis (**b**). *n* = 4. Scale bar, 50 μm. **C** CK-MB and cTnT concentrations in rat serum. *n* = 5. Data were expressed as mean ± SD. ^*^p < 0.05 *vs* control

Hematoxylin-eosin (H&E) staining showed an irregular arrangement of myocardial fibers and distinct inflammatory cell infiltration in rat heart tissues after SiNP exposure (Fig. 2A-a). Correspondingly, the histopathological score of heart tissues indicated a dose-dependent myocardial injury (Fig. 2A-b). Masson’s trichrome staining (Fig. 2B-a) and corresponding collagen volume fraction analysis (Fig. 2B-b) demonstrated that the exposure to SiNPs may facilitate collagen proliferation and deposition in the myocardial interstitium. Concomitantly, the serum levels of creatine kinase-MB (CK-MB) and cardiac troponin T (cTnT) as specific indicators for myocardial injury, were remarkably elevated after SiNPs instillation (Fig. 2C).

### SiNPs triggered ferroptosis in rat cardiac tissues in vivo

**Fig. 3:**
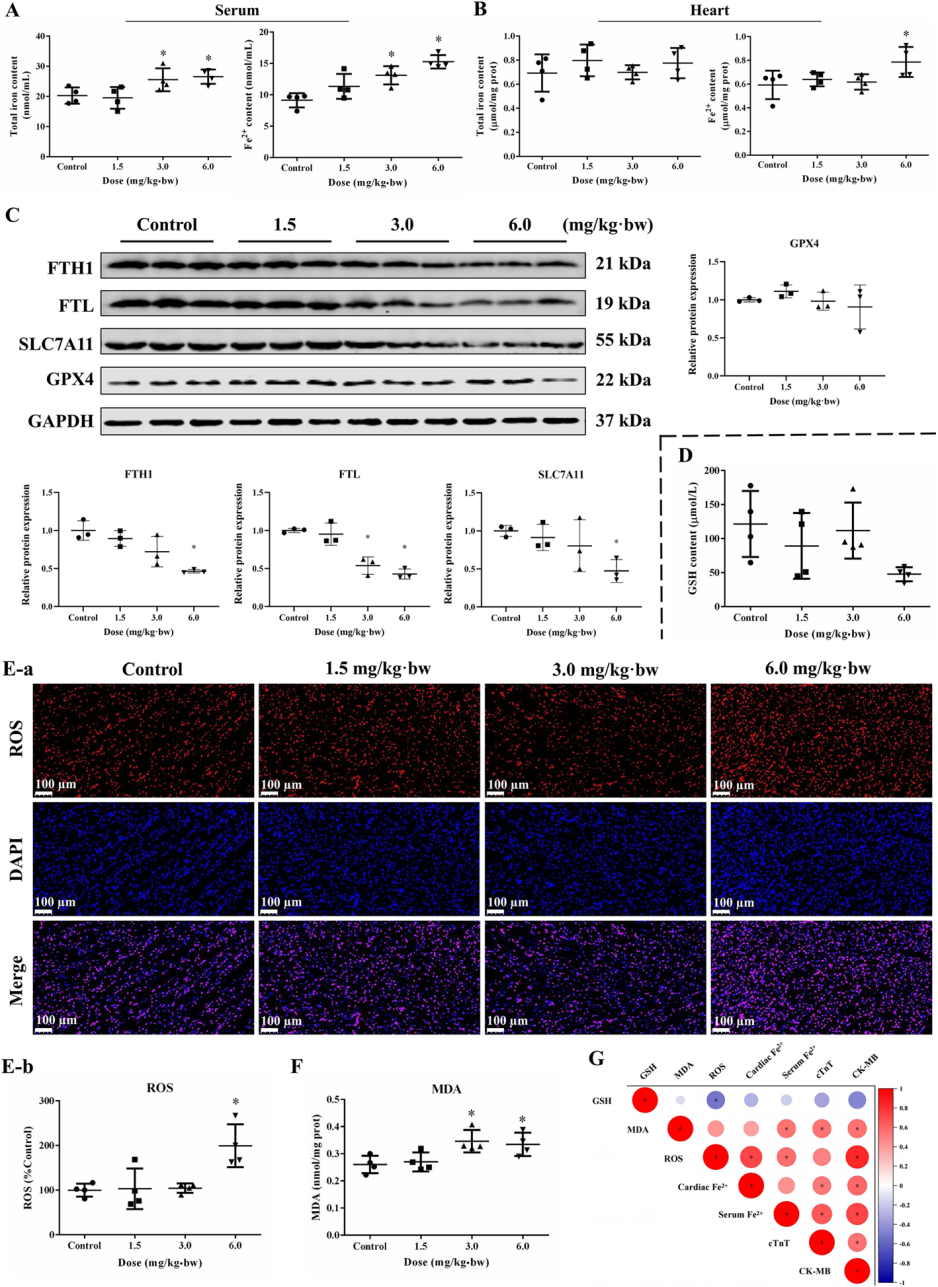
SiNPs induced iron overload and lipid peroxidation in rat hearts. Total iron and Fe^2+^ contents in rat serum (**A**) and heart tissues (**B**). *n* = 4. **C** Protein expressions in rat heart tissues. *n* = 3. **D** GSH content in serum. **E** Immunofluorescence of ROS (**a**) and corresponding fluorescence intensity analysis (**b**). *n* = 4. Scale bar, 100 μm. **F** MDA content in rat hearts. *n* = 4. **G** Correlation analysis of the indicators in rats. *n* = 4. Data were expressed as mean ± SD. ^*^p < 0.05 *vs* control

Fe^2+^ is the key substrate of the Fenton reaction, and the break of iron balance is one of the major triggers of ferroptosis. Firstly, the total Fe and Fe^2+^ contents in the serum and heart tissues of rats were measured. As a result, the total iron and Fe^2+^ in the serum were distinctly elevated after SiNPs exposure (Fig. 3A). Meanwhile, a cardiac Fe^2+^ overload was manifested upon SiNPs exposure at a higher dose (6.0 mg/kg·bw; Fig. 3B). Western blot assay (Fig. 3C) showed the intratracheal instillation of SiNPs down-regulated the levels of ferritin heavy chain 1 (FTH1) and ferritin light chain (FTL), hinting the disturbed iron metabolism in rat hearts. Solute carrier family 7 member 11 (SLC7A11) is an important component of system Xc- to regulate glutathione (GSH) biosynthesis and ensuing ferroptosis. In comparison to the control, the down-regulated SLC7A11 in heart tissues and GSH in serum was seen along with the increased exposure dosage of SiNPs (Fig. 3C and D). Also, the decreasing trend of cardiac GPX4 was detected in SiNPs-exposed rats (Fig. 3C). In support of the activation of lipid peroxidation, a large amount of ROS was detected to be accumulated in heart tissues upon SiNPs exposure (Fig. 3E), accompanied by a prominent elevation of malondialdehyde (MDA) content in cardiac tissues by SiNPs (Fig. 3F). All these data revealed SiNPs could trigger ferroptosis in rat cardiac tissues, as evidenced by Fe^2+^ overload, imbalanced iron metabolism and lipid peroxidation. Of note, the correlation analysis between myocardial and serum indexes of rats well explained ferroptosis may serve as a key player in the induction of myocardial injury by SiNPs (Fig. 3G). The cardiac Fe^2+^ overload, elevated ROS and MDA content were all positively correlated to the myocardial injury (as reflected by CK-MB or cTnT). Also, the Fe^2+^ overload was positively correlated to ROS accumulation in the rat heart.

### SiNPs induced ferroptosis in in vitro cultured cardiomyocytes

**Fig. 4:**
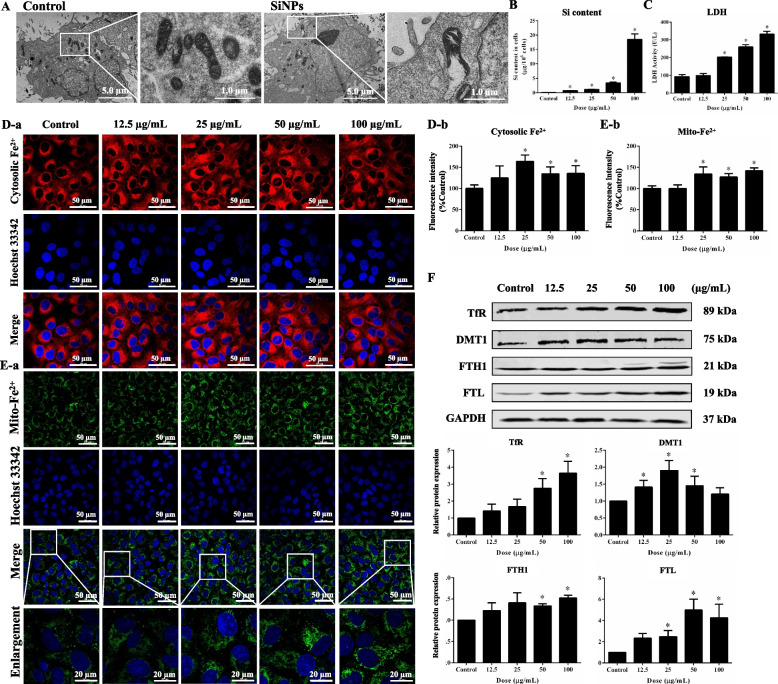
SiNPs induced cellular damage and iron overload in cardiomyocytes. AC16 cells were incubated with SiNPs for 24 h. **A** TEM images upon SiNPs stimuli (50 μg/mL, 24 h). Scale bar, 1.0 or 5.0 µm **B** Intracellular silicon content. **C** LDH activity. **D** Cytosolic Fe^2+^ fluorescent labeling (**a**) and corresponding analysis (**b**). Scale bar, 50 µm. **E** Mitochondrial Fe^2+^ levels (**a**) and relative analysis (**b**). Scale bar, 20 or 50 µm. **F** Western blot assay. Data were expressed as mean ± SD. *p < 0.05 *vs* control

The dosage of SiNPs in vitro was based on cell viability measurement upon SiNPs treatment for 24 h. A dose-dependent decline in cell viability was manifested (Fig. S1A). Of note, the cell viability was significantly reduced to about 70% at the concentration of 100 µg/mL, which was set as the highest dosage. In comparison to the control, no significant difference was seen in the 12.5 µg/mL group, setting as the lowest dosage. Of note, the applied dosage of SiNPs (12.5-100 µg/mL) in vitro was corresponding to 2.27-18.18 µg/cm^2^. As manifested in TEM images (Fig. 4A), SiNPs can enter into the cytoplasm of AC16 cells, resulting in severely damaged mitochondria as evidenced by the mitochondrial membrane rupture and cristae disappearance. Consistently, a dose-dependent intracellular Si content was significantly elevated in AC16 cells after SiNP treatment (Fig. 4B). Concomitantly, an increase in lactate dehydrogenase (LDH) release was noticed after SiNP exposure (Fig. 4C), hinting the collapse of cellular membrane. In addition, LDH release was correlated to the declined cell viability induced by SiNPs (Fig. S1B).

To realize the effects of SiNPs on iron metabolism in AC16 cells, we separately labeled Fe^2+^ in cytosol and mitochondria with specific fluorescent probes, FerroOrange and Mito-FerroGreen. As a result, the fluorescence images (Fig. 4D-a) and semi-quantitative analysis (Fig. 4D-b) proved the induction of cytosolic Fe^2+^ overload in AC16 cells by SiNPs. Consistently, mitochondrial Fe^2+^ levels were also greatly enhanced upon SiNP exposure (Fig. 4E). Further, the mRNA and protein expressions of molecules related to cellular iron metabolism were examined. In the iron metabolism pathways, ferric iron (Fe^3+^) is transferred into the cell by transferrin receptor (TFRC/TfR), then converted into Fe^2+^ in the endosome and released from the endosome by divalent metal transporter (DMT1). Fe^2+^ is stored in a labile iron pool (LIP) and ferritin (composed of FTH1 and FTL). Results showed that upon SiNPs stimuli, TfR, FTH1, FTL, and DMT1 were all remarkably up-regulated (Fig. 4F). Concertedly, the transcriptional levels of *TFRC*, *FTH1*, *FTL*, and *DMT1* genes were elevated after SiNPs treatment (Fig. S2).

**Fig. 5:**
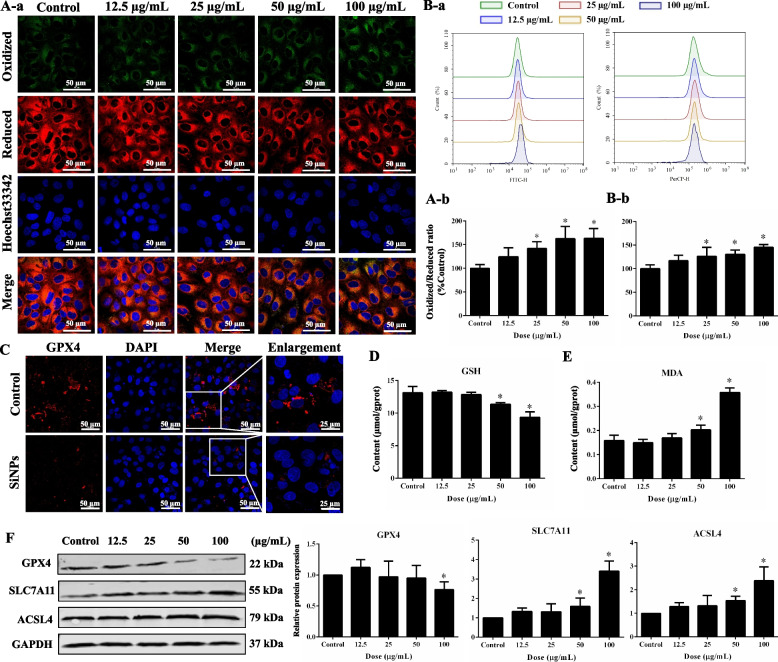
SiNPs caused lipid peroxides accumulation in cardiomyocytes. **A** The cells were subjected to C11-BODIPY^581/591^, and imaged (**a**), correspondingly semi-quantified to calculate oxidized/reduced ratio (**b**). Scale bar, 50 µm. **B** The fluorescent intensity of cells labeled by C11-BODIPY^581/591^ was also measured using flow cytometry (**a**), and oxidized/reduced ratio was calculated for lipid ROS quantification (**b**). **C** GPX4 measurement by immunofluorescence. Scale bar, 25 or 50 µm. **D** GSH contents. **E** MDA contents. **F** Western blot assay. Data were expressed as mean ± SD. ^*^p < 0.05 *vs* control

Lipid ROS acts as the end product of the Fenton reaction. As shown in Fig. 5A, enhanced oxidation state (green fluorescence) and declined reduction state (red fluorescence) were observed in C11-BODIPY^581/591^-labeled AC16 cells after SiNPs treatment. The fluorescent quantification by flow cytometry (FCM) analysis revealed a dose-dependent increment of lipid ROS caused by SiNPs (Fig. 5B). SiNPs triggered extensive recession of the cellular GPX4 (Fig. 5C), which was further confirmed by gene and protein measurements (Fig. 5F and Fig. S2). Moreover, the exposure to SiNPs led to a reduced GSH (Fig. 5D) and increased MDA (Fig. 5E) in AC16 cells. Acyl-CoA synthetase long-chain family member 4 (ACSL4) is a key marker of lipid peroxidation in ferroptosis. ACSL4 and SLC7A11 were both significantly elevated after SiNPs administration (Fig. 5F and Fig. S2), indicating the presence of lipid peroxidation and the imbalance of cellular antioxidant system. All these findings suggested that SiNPs disturbed the iron metabolism balance, resulting in Fe^2+^ overload, ensuing Fe^2+^-related lipid peroxidation, and the resultant ferroptosis in cardiomyocytes.

**Fig. 6:**
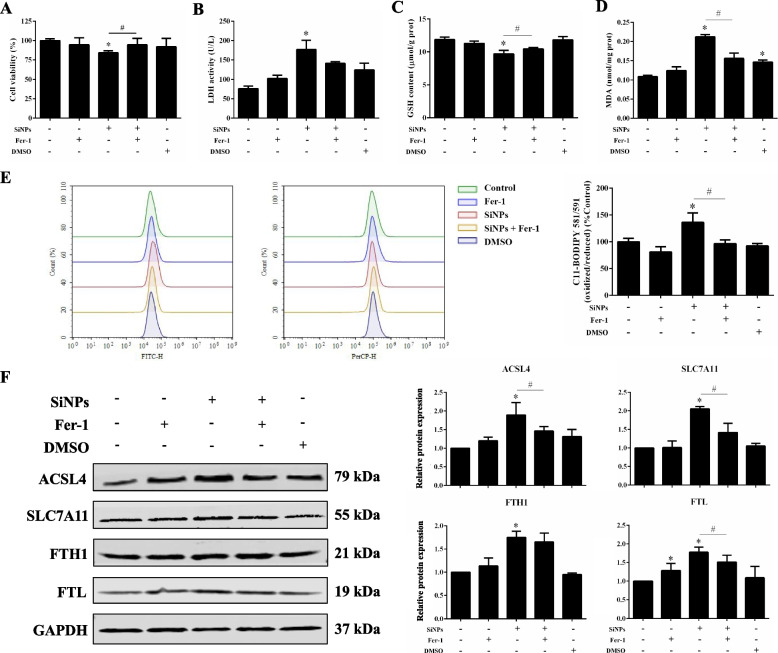
Fer-1 alleviated SiNPs-elicited ferroptosis in cardiomyocytes. **A** Cell viability, **B** LDH activity, **C** GSH, and **D** MDA contents were measured in AC16 cells after Fer-1 and SiNPs treatment. **E** Through C11-BODIPY^581/591^ labeling, the cellular level of lipid ROS was quantified by FCM. **F** Western blot analysis. Data were expressed as mean ± SD. ^*^p < 0.05 *vs* control, ^#^*p* < 0.05 *vs* SiNPs

To validate the role of ferroptosis in SiNP-elicited myocardial cytotoxicity, we treated AC16 cells with Fer-1 before SiNP exposure in vitro. The results presented Fer-1 significantly ameliorated the declined cell viability caused by SiNPs (Fig. 6A), as well as slightly reversed the increased LDH release (Fig. 6B). Moreover, Fer-1 could remarkably alleviate the lipid peroxidation caused by SiNPs, as evidenced by the intracellular GSH, MDA and lipid ROS measurements (Fig. 6C-E). In parallel, Fer-1 could significantly inhibit SiNPs-induced up-regulation on expressions of ACSL4, SLC7A11 and FTL, illustrating the alleviation of iron overload, and inactivation of lipid peroxidation caused by SiNPs (Fig. 6F). Overall, these data reflected the pivotal role of ferroptosis in cardiomyocyte injury upon SiNPs stimuli.

### SiNPs activated HO-1 to mediate ferroptosis

**Fig. 7:**
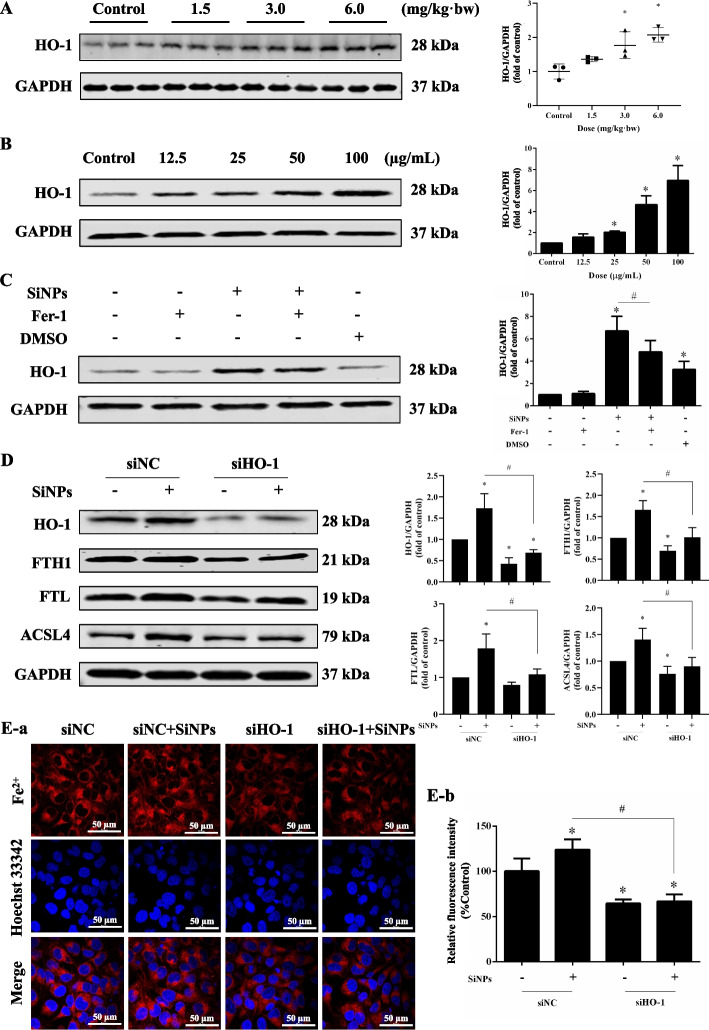
HO-1 as a crucial player in SiNPs-induced ferroptosis of cardiomyocytes. HO-1 expressions in SiNPs-exposed rat hearts (**A**) and AC16 cells (**B**). **C** Fer-1 inhibited the elevation of HO-1 by SiNPs in AC16 cells. Cells were incubated with siHO-1 and SiNPs (50 μg/mL, 24 h) to verify the role of HO-1 in SiNPs-induced ferroptosis. **D** Western blot analysis. **E** Cytosolic Fe^2+^ level determination using FerroOrange. The fluorescent image (**a**) was captured, and corresponding fluorescence intensity was analyzed (**b**). Scale bar, 50 µm. Data were expressed as mean ± SD. ^*^p < 0.05 *vs* control, ^#^*p* < 0.05 *vs* SiNPs

Our previous study stated that SiNP exposure could significantly upregulate the expression of HO-1 in rat aortas [31] and in vitro cultured vascular endothelial cells [20]. Here, HO-1 activation was presented in both rat hearts (Fig. 7A) and in vitro cultured AC16 cells (Fig. 7B) upon SiNPs stimuli, which could be reversed by Fer-1 (Fig. 7C). Higher expression of HO-1 was associated with the severity of coronary heart disease [6]. Apart from a critical oxidant-sensitive regulator, HO-1 could catalyze the region-specific hydroxylation of heme to ferrous iron, carbon monoxide, and biliverdin. A growing body of research has illuminated that HO-1 could serve as a critical player in ferroptosis by modulating Fe^2+^ production and iron-dependent lipid peroxidation [28, 34, 65], and contribute to myocardial injury by inducing ferroptosis [26, 45, 46]. To forcefully test the effect of HO-1 on ferroptosis, we transfected AC16 cells with synthesized siHO-1 sequences. As manifested in Fig. S3A, siHO-1 was successfully transfected into AC16 cells for 12 h and evenly dispersed. The sequence of siHO-1, especially siHO-1(2), was verified to possess inhibitory effects on HO-1 transcription (Fig. S3B), and used for further validation. HO-1 inhibition by siHO-1 could effectively lower the up-regulated HO-1, ACSL4, FTH1, and FTL caused by SiNPs (Fig. 7D), as well as weakening the cytosolic Fe^2+^ overload (Fig. 7E). All these findings illustrated that HO-1 may serve as a critical regulator for cardiomyocytes ferroptosis caused by SiNPs through modulating iron production and lipid peroxidation.

### miR-125b-2-3p mediated cardiomyocytes ferroptosis by targeting HO-1

**Fig. 8:**
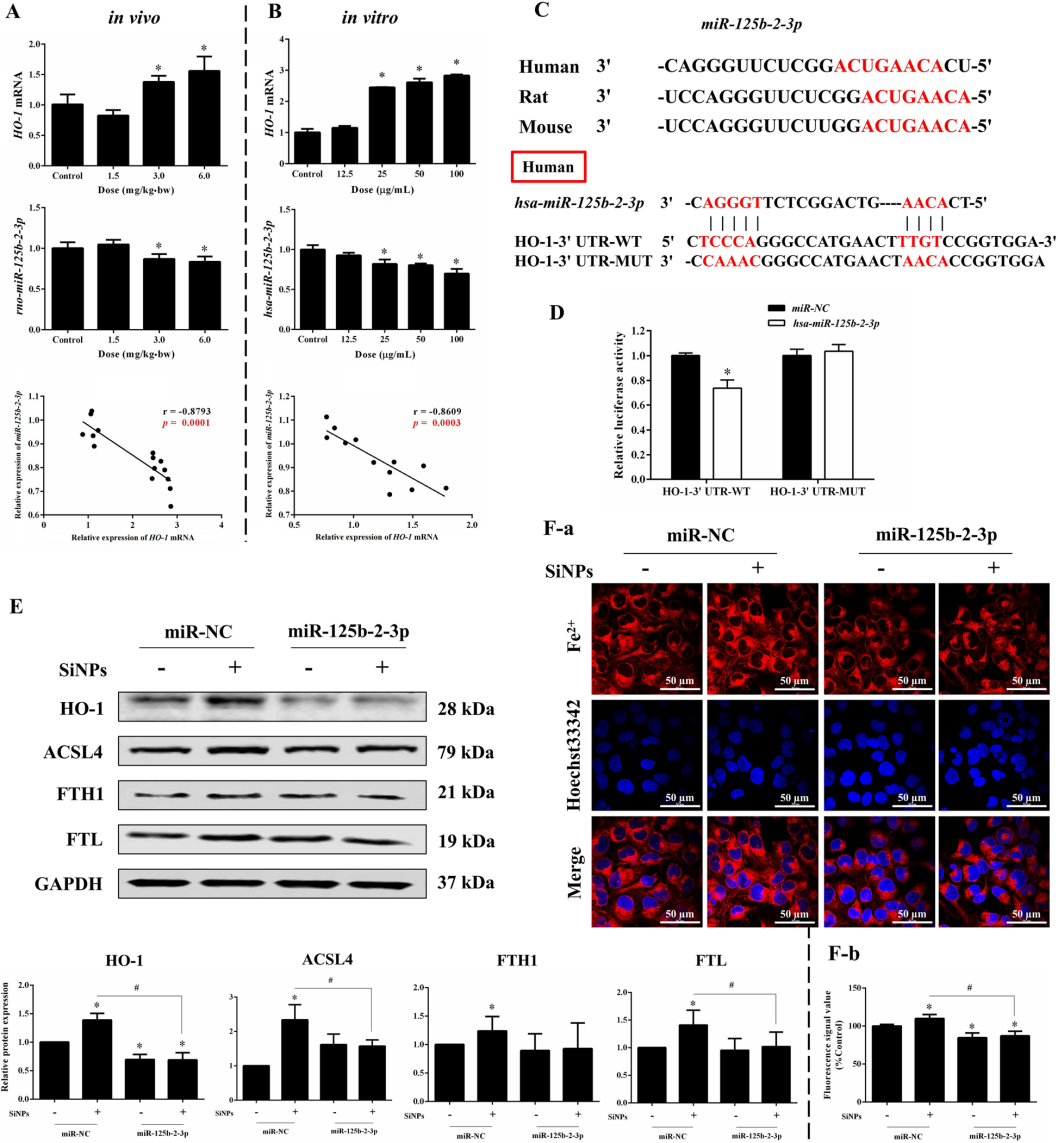
miR-125b-2-3p targeted HO-1 to mediate ferroptosis triggered by SiNPs. **A**-**B** RT-PCR determination and correlation analysis of HO-1 mRNA and miR-125b-2-3p levels upon SiNPs exposure in vivo and in vitro. **C** Gene sequences of miR-125b-2-3p, and binding sites between miR-125b-2-3p and the 3’-UTR of HO-1. and its connection sites with HO-1. **D** Relative luciferase activity. miR-125b-2-3p mimics (50 nM) or miR-NC was transfected in AC16 cells, which were then treated with SiNPs (50 μg/mL, 24 h). Western blot analysis **E**, the cytosolic Fe^2+^ labeling (**F**-**a**), and its corresponding analysis (**F**-**b**) were performed. Scale bar, 50 µm. Data were expressed as mean ± SD. ^*^p < 0.05 *vs* control, ^#^*p* < 0.05 *vs* SiNPs

According to our previous heart microarray analysis in a rat model of SiNP exposure via intratracheal instillation, miR-125b-2-3p was screened out as one of the important differentially expressed miRNA in the rat heart tissues upon SiNP exposure, and predicted to bind the 3’UTR of HO-1. In this context, we choose miR-125b-2-3p to study whether it regulated HO-1 transcription and resultant ferroptosis in cardiomyocytes upon SiNP stimuli. As depicted in Fig. 8A and B, miR-125b-2-3p was dose-dependently declined in SiNPs-exposed cardiac tissue in vivo and in vitro cultured AC16 cells. Conversely, HO-1 mRNA was notably elevated, and negative correlations to miR-125b-2-3p were noticed (Fig. 8A and B). Notably, the sequences of miR-125b-2-3p were highly conserved (Fig. 8C). Double luciferase assay verified the targeted binding site of hsa-miR-125b-2-3p and HO-1 (Fig. 8C and D), indicating that miR-125b-2-3p could directly target HO-1 to regulate its transcription.

Given the critical role of HO-1 in myocardial ferroptosis above mentioned, the miR-125b-2-3p mimics were utilized to figure out the involvement of miR-125b-2-3p. As expected, the overexpressed miR-125b-2-3p (Fig. S3C) significantly declined HO-1 level in AC16 cells, and greatly reversed SiNPs-induced HO-1 activation (Fig. 8E). Moreover, miR-125b-2-3p lowered the expressions of ACSL4 and iron storage proteins (FTH1 and FTL) in comparison to SiNPs group (Fig. 8E). Besides, miR-125b-2-3p overexpression significantly inhibited intracellular Fe^2+^ overload induced by SiNPs (Fig. 8F). All these data suggested SiNPs may induce cardiac ferroptosis through miR-125b-2-3p-targeting HO-1 signaling, which was responsible for the iron overload and lipid peroxidation.

## Discussion

At present, ferroptosis has been reported to participate in some NP-elicited deleterious cardiac effects [78]. Nevertheless, its involvement is still unknown upon SiNP exposure, let alone the corresponding mechanisms. Iron is the most abundant transition metal element in living organisms, which participates in various physiological processes, e.g., oxygen and lipid metabolism, cellular respiration, and DNA synthesis. Oppositely, iron also has potential toxicity, and participates in the pathological process of cardiovascular disease [26]. Research has suggested that the severity of myocardial injury is proportional to the magnitude of cardiac iron overload [18, 50]. Iron accumulation has been demonstrated as a key mediator of impaired redox homeostasis and ultimate cell death [43]. Excessive iron accumulation in the heart leads to reduced cardiac function, impaired mitochondrial dynamics, and mitochondrial dysfunction, contributing to the loss of cardiomyocytes [55]. Intriguingly, iron metabolism in human and experimental animals is gender dependent. In comparison to males, female mice are markedly protected from iron-induced cardiomyopathy, i.e., myocardial dysfunction and fibrosis, probably due to the ovarian secretion of 17-β-estradiol [8]. Thereby, male Wistar rats were preferentially selected. Consequently, the respiratory exposure of SiNPs via intratracheal instillation led to iron enrichment in both serum and heart tissues, particularly Fe^2+^ (Fig. 3A and B). Of note, iron overload was a critical contributor to the induction of myocardial injury by SiNPs, which was also positively correlated with oxidative stress and lipid peroxidation (Fig. 3G).

In recent years, intracellular free iron ions have attracted more and more attention due to their high reactivity and association with cell damage and death. Free iron usually presents in a stable redox state in the form of Fe^2+^ and Fe^3+^. Comparatively, the intracellular Fe^2+^ activity is more important than Fe^3+^ given ion water-solubility and the reduction environment in cells. Consistently, Shu et al*.* reported that Fe^2+^ but not Fe^3+^ accumulation could elevate the levels of oxidative stress indicators and ferroptosis-related molecules in the retina [52]. Therefore, the cytosolic Fe^2+^ level is a key indicator for iron death. In line with the in vivo findings, cytosolic Fe^2+^ in SiNPs-treated cardiomyocytes was significantly increased, as well as mitochondrial Fe^2+^ (Fig. 4D and E). The observed iron overload could be well explained by the up-regulated TfR and DMT1 to facilitate iron entry and transportation (Fig. 4F and Fig. S2). The upregulation of TfR could significantly increase the intracellular iron load and enhance cell sensitivity to GPX4-induced ferroptosis [40]. DMT1 is proven to promote TfR-mediated iron uptake and dietary iron absorption [3], and its overexpression could significantly promote ferroptosis of cardiomyocytes in acute myocardial infarction [53].

LIP is regulated by ferritin which is composed of FTH1 and FTL [1]. According to the literature [13, 68], ferritin holds a negative regulatory role in ferroptosis, which protects against cardiac ferroptosis and subsequent heart failure. Excessive Fe^2+^ in LIP binds with ferritin to store iron, which would be released when cells are iron deficient. Overactivation of selective autophagy degraded ferritin for the release of iron, resulting in iron-mediated ferroptosis in cardiomyocytes [29]. In addition, ferritin catalyzes the oxidation of Fe^2+^ at the ferroxidase center to prevent free Fe^2+^ from producing oxygen free radicals through the Fenton reaction [75]. Abnormal changes in ferritin subunit composition (FTH1 and FTL) might affect iron uptake and storage. Interestingly, FTH1 and FTL were significantly down-regulated in Wistar rat heart tissues (Fig. 3C) after SiNPs exposure, on the contrary, their expression in mRNA and protein levels were up-regulated in SiNPs-treated AC16 cells (Fig. 4F and Fig. S2). This phenomenon may be attributed to the exposure mode difference between in vivo (sub-chronic long exposure) and in vitro (an acute 24-h exposure) models. To verify our hypothesis, we conducted a continuous exposure of SiNPs in vitro for 10 passages in AC16 cells. Interestingly, the increased expressions of FTH1 and FTL by SiNPs were lowered down to the control level in 5th passage (P_5_) cells, and were significantly decreased in P_10_ cells (Fig. S4). In this context, the up-regulated ferritin may be interpreted as increased stress after iron overload. In line with our finding, elevated expression of FTH1 was noticed in a study of PM_2.5_-induced ferroptosis of endothelial cells, which led to iron storage dysfunction and ferroptosis by disrupting the cellular antioxidant defense system [67]. Besides, significantly upregulated mRNA levels of FTH1 and FTL were induced by erastin (a classical ferroptosis inducer) [56].

Iron-mediated oxidation and lipid peroxidation are fundamental for ferroptosis. Once the balance of iron metabolism (iron uptake, utilization, storage, and recycling) was disrupted, the accumulated free iron ions might trigger the Fenton reaction, followed by the activation of lipoxygenases (LOX), the generation of lipid peroxides, and the resultant ferroptosis [58, 71]. A few indicators for oxidative stress and lipid peroxidation have been detected among the SiNPs-induced myocardial toxicity investigations, e.g., elevated ROS and MDA, whilst loss in antioxidant ability [48, 63]. Oxidative stress and lipid peroxidation were clearly seen in SiNPs-exposed rat heart and cardiomyocytes (Figs. 3 and 5). Importantly, the lipid peroxidation induced by SiNPs was reversed by Fer-1 (Fig. 6), illustrating the cardiomyocyte ferroptosis caused by SiNPs was attributable to lipid peroxide accumulation. ACSL4 is an important biomarker of ferroptosis, and its formation activates the oxidation of unsaturated fatty acids on the cell membrane, causing a large number of lipid peroxidation products in cells [11]. As the important isoenzyme in the metabolism of polyunsaturated fatty acids (PUFA), ACSL4 determined the sensitivity of ferroptosis. Certainly, ACSL4-mediated ferroptosis was found to be involved in the sensitivity regulation of cardiac remodeling and contraction [47]. In line with the excessive ROS generation and MDA production, ACSL4 expression was enhanced after SiNPs exposure (Fig. 5).

GSH biosynthesis and GPX4 function as important components in maintaining redox homeostasis, and were known as negative regulators for ferroptosis through inhibiting lipid peroxidation. Tang et al*.* reported the inactivation of GPX4 and collection of intracellular lipid peroxides mediated by GSH consumption were the main causes of ferroptosis [59]. Ferroptosis in endothelial cells induced by zinc oxide NPs was closely related to intracellular GSH depletion and GPX4 downregulation [74]. In agreement with the downward trend in GSH and cardiac GPX4 expression in vivo (Fig. 3C-D), GPX4 and GSH in AC16 cells were significantly declined by SiNPs (Fig. 5D and F), suggesting the destroyed antioxidant system of cardiomyocytes by SiNPs. System Xc^−^ introduces cystine as a key substrate for the synthesis of GSH, which is associated with GPX4 to antagonize ferroptosis [54]. SLC7A11 mainly functions for amino acid transport and represents the specificity of system Xc^−^. The inhibition of SLC7A11 led to depressed cystine uptake and disordered GSH synthesis. Intriguingly, the inconsistent data related to SLC7A11 expression was noticed in the in vivo and in vitro models. The increased SLC7A11 in SiNPs-treated AC16 cells was probably a negative feedback regulator to oxidative stress. The pharmacological inhibition of systemic Xc^−^ in tumor cells demonstrated a compensatory increase in SLC7A11 expression, similar compensatory elevation was also induced by erastin while GSH content was declined [10]. Interestingly, studies have stated SLC7A11 as the downstream gene of Nrf2, and the up-regulated Nrf2 in ferroptosis was accompanied by the elevated SLC7A11 [16, 49]. In parallel to up-regulated SLC7A11 (Fig. 5F and Fig. S2), Nrf2 was activated by SiNPs in cardiomyocytes (Fig. S5). In this context, we speculated the up-regulated SLC7A11 caused by SiNPs as a negative feedback mechanism in response to oxidative stress.

In particular, HO-1 was recognized as the central regulator of iron homeostasis, apart from its role in antioxidation regulation. In a physiological state, HO-1 can catalyze the catabolism of heme to produce Fe^2+^. Under aging and inflammatory stimulation, the high expression of HO-1 was often accompanied by changes in iron metabolizing proteins, resulting in growing iron deposition, oxidative stress, and ferroptosis [17]. In doxorubicin (DOX)-induced cardiomyopathy, the upregulated HO-1 caused apparent heme degradation and free iron release in the myocardium, leading to ferroptosis and ultimate heart failure. Inhibiting HO-1, or iron chelation remarkably prevented ferroptosis in cardiomyocytes, and greatly alleviated DOX-induced cardiac injury and heart failure [57]. Our previous studies found that SiNPs-induced cytotoxicity was accompanied by altered Nrf2/HO-1 signaling [20, 31, 76]. Studies have revealed the activation of oxidative stress can induce a nuclear translocation of Nrf2 to regulate an increased HO-1 expression, thereby leading to LIP overload and ferroptosis [7, 69]. Overactivation of HO-1 could disrupt the balance of iron metabolism [5, 60], and inhibition of HO-1 significantly improved cell viability and reduced intracellular iron accumulation [30]. In line with these findings, our data presented HO-1 as a crucial regulator for iron overload and the resultant lipid peroxidation and myocardial ferroptosis (Fig. 7). More importantly, miR-125b-2-3p could target HO-1 to participate in the ferroptosis procession caused by SiNPs in cardiomyocytes (Fig. 8), i.e., iron overload and lipid peroxidation.

miRNAs are important regulators of cardiac physiological and pathological conditions and potential biomarkers for the diagnosis and prevention of cardiac diseases. The miR-125 family is highly conserved and involved in the process of myocardial pathological injury, including miR-125a, miR-125b-1, and miR-125b-2 [66]. miR-125b is highly expressed in myocardial tissue and participates in regulating the cell biological processes, e.g., proliferation, differentiation, and death of myocardial cells. Studies have confirmed miR-125b as a potential intervention target for the prevention and treatment of heart diseases [61]. Decreased miR-125b expression promoted ROS generation in cardiomyocytes, exacerbated oxidative stress, and led to myocardial dysfunction [73], while overexpressed miR-125b inhibited myocardial injury [38]. However, only a few studies have examined the relationship between miR-125 family members and ferroptosis. Here, our study first proposed miR-125b-2-3p-targeted HO-1 as a key regulatory mechanism in the induction of cardiomyocyte ferroptosis by SiNPs. Certainly, we don’t exclude other miRNAs in the regulation of ferroptosis and the consequent cardiac toxicity. For instance, the ectopic expression of miR-137 inhibited SLC1A5 to promote ferroptosis, leading to reduced glutamine uptake and increased MDA production [41]. miR-351-5p targeted MLK3 to restrain ferroptosis, leading to the improvement in mice’s cardiac function [64].

Certainly, this work has also initiated some ideas for our future research. Firstly, the causal relationship between ferroptosis and myocardial injury should be further validated in vivo. Secondly, from the perspectives of ROS-mediated lipid peroxidation as an important event in the process of cell death, e.g., apoptosis, ferroptosis, and autophagy [62], their connectivity and possible crosstalk in the pathological myocardial cell death is of great concern. Several studies have suggested that ferroptosis may occur prior to apoptosis and promote cellular susceptibility to apoptosis, and that apoptosis could be converted to ferroptosis under certain conditions [70]. As manifested in Fig. S6, Fer-1 pretreatment in cardiomyocytes could greatly block the incidence of apoptosis caused by SiNPs, hinting at ferroptosis as a requisite player on myocardial apoptosis upon SiNP stimuli, ultimately leading to myocardial diseases. Thirdly, the underlying mechanistic investigations into SiNPs-elicited ferroptosis in cardiomyocytes remain in need, which would help to unveil novel strategies for the prevention and control of SiNP-elicited myocardial toxicity.

## Conclusion

In summary, our present study first confirmed the induction of cardiomyocyte ferroptosis as a key contributor to myocardial injury by SiNPs in vivo and in vitro, which was characterized by iron (Fe^2+^) overload, lipid peroxidation, and GSH/GPX4 depletion. More importantly, the mechanistic investigation revealed that miR-125b-2-3p-targeted HO-1 was responsible for SiNPs-induced ferroptosis through modulating iron metabolism, leading to Fe^2+^ overload and lipid peroxidation in cardiomyocytes. Of note, HO-1 inhibition or Fer-1 may be a practicable measure for resisting myocardial toxicity caused by SiNPs. It’s worth mentioning that cardiovascular health risks of prolonged SiNP exposure would persist even at a relatively low exposure level. Certainly, a deep understanding of SiNPs-induced potential adverse effects will facilitate the design of safer silica-related nanomaterials and the development of more efficient nanomedicine for disease diagnosis and treatment.

## Materials and methods

### Particles preparation

Amorphous SiNPs applied in this work were acquired by using the Stöber approach as described previously [21]. Lastly, the stock solution for SiNPs dispersed in deionized water was prepared by 5-min sonication (160 W, 20 kHz) in a Bioruptor UCD-200, Belgium, and 20-min sterilization (0.1 MPa, 120 °C). The particle concentration in suspension was calculated by weighing after freeze-drying. Further, the shape and size of particles were detected using TEM (JEOL JEM2100, Japan) and analyzed by using Image J software. Moreover, the hydrodynamic size and zeta potential of SiNPs in culture medium (DMEM/F12; Biological Industries, Israel) and physiological saline (0.9%) for 24 h were measured through transmission light scattering method in a Malvern Zetasizer (Nano ZS90, UK).

### Animal studies

Thirty-two male Wistar rats (6-week-old, Vital River, China) were housed in the Experimental Animal Center, Capital Medical University in a specific pathogen-free (SPF) condition: temperature 24 ± 1℃, humidity 50 ± 5%, a 12 h light-dark cycle. All rats were provided with the certified rodent diet and sterilized tap water ad libitum. After acclimation, the rats were randomly divided into four groups (one control and three SiNP groups: 1.5, 3.0, or 6.0 mg/kg·bw via intratracheal instillation, once per week for 12 times). The control rat was administered with physiological saline instead. The instillation volume per rat was controlled at 200 ± 20 µL. Ultimately, all rats were sacrificed one week after the last particle instillation, and blood samples and heart tissues were harvested for further examination. All procedures were in concert with the requirements of Experimental Animals Care and Use of Capital Medical University (Ethical number, AEEI-2019-177).

According to the literature [42, 72], the SiNPs exposure dosage was set based on the permissible concentration of amorphous SiO_2_ (5 mg/m^3^) in the workplace, physiological parameters, and conversion coefficient. Given NPs administered once per week, the applied highest dosage (6.0 mg/kg·bw) is approximately equivalent to the lung burden of SiNPs after a workweek (40 h) breath in air containing 1.2 mg/m^3^ amorphous silica. In this context, we establish a relatively low-level, sub-chronic exposure model of SiNPs in rats.

### Histopathology and immunofluorescence

Heart tissues were fixed with 4% paraformaldehyde and embedded in paraffin blocks, as well as an optimal cutting temperature (OCT) compound. Then, 5 µm thick slices fixed on the slide were stained with H&E (Servicebio, China) and Masson’s trichrome solution (Servicebio, China) for pathological injury assessment, stained with dihydroethidium (DHE) for ROS fluorescent labeling. All slides were recorded through Pannoramic Digital Slide Scanner and analyzed by CaseViewer (3DHISTECH, Hungary) or Image J software (NIH, USA). Afterward, the myocardial injury was scored in light of the degrees and scope of histopathological lesions in the tissue sections [23, 48]. The percentage of positive area and total tissue area in the full field was measured for semi-quantitative analysis of collagen hyperplasia and ROS accumulation.

### Myocardial injury assessment

The rat blood sample was harvested, and the serum was acquired through centrifugation at 3000 rpm, 4℃ for 10 min. Markers of myocardial injury in serum were quantified by ELISA kits for CK-MB (Jiancheng, China) and cTnT (Cloud-Clone, China). The contents of reduced GSH in serum and MDA in heart tissues were measured according to the manual protocols (Jiancheng, China).

### Iron assay

The total iron ion and Fe^2+^ contents in both the serum and heart tissues of rats were measured through an iron assay kit (Abcam, UK). Briefly, heart tissues in each group were homogenized with assay buffer, then the supernatants were obtained after centrifugating at 1600 g for 10 min. Serum or supernatant samples were incubated with assay buffer for Fe^2+^ assay. Then for the acquisition of total iron ions in samples, all Fe^3+^ were reduced to form Fe^2+^ by incubating with an iron reducer. After incubating with the iron probe for 1 h, the contents for either Fe^2+^ or total iron ion were quantified through a microplate reader (BioTek, USA) at 593 nm.

### Cell culture and treatment

AC16 acquired from Cell Resource Center, Shanghai Institutes for Biological Sciences, China, were routinely cultured in DMEM/F-12 with 1% Penicillin-Streptomycin solution (KeyGEN, China) and 10% fetal bovine serum (FBS; Gibco, USA) in a 5% CO_2_ incubator at 37℃. After growing adherent with about 80% confluency, the cells were incubated with SiNPs solution diluted by the serum-free DMEM/F-12 medium at the indicated doses (12.5, 25, 50, and 100 µg/mL, respectively) for 24 h. Instead, the control cells were treated with equal volume serum-free medium. The dosage of SiNPs in this study was selected following our previous in vitro study [77].

For the mechanism investigations, the cells were preincubated with Fer-1 (1.25 µM; Sigma-Aldrich, USA) for 2 h before SiNPs treatment (50 µg/mL). Moreover, HO-1 siRNA (siHO-1), miR-125b-2-3p mimics (50 nM; OligoBio, China) or the corresponding negative control (named siNC and miR-NC, respectively) were transfected into cells by using RNA Trans Mate (Sangon Biotech, China) for 12 h and then exposed to SiNPs (50 µg/mL) for 24 h. The corresponding sequences are listed in Table S2.

### Cell viability and LDH assay

A Cell Counting Kit-8 (CCK-8; LabLead, China) was applied to determine cell viability after a 24-h SiNPs treatment for toxicity assessment. Besides, LDH would be directly released into the culture medium when the cell membrane integrity was ruptured. Thus, the cell culture supernatants were harvested after SiNP exposure, and the LDH activity was measured by using its corresponding kit following the product manual (Jiancheng, China).

### SiNPs uptake and TEM observation

As described previously [21], cells were collected after SiNP treatment, and cracked by HNO_3_ and H_2_O_2_ (3:1). The Si content in cell lysate was measured by the inductively coupled plasma-atomic emission spectrometry (ICP-AES; Agilent 720; Agilent Technologies, USA). Moreover, the alterations of cell morphology and organelle structure were analyzed by TEM (JEM2100, JEOL, Japan) after SiNP exposure (50 µg/mL, 24 h).

### Fe^2+^ label

FerroOrange (1 µM; DOJINDO, Japan) and Mito-FerroGreen (5 µM; DOJINDO, Japan) fluorescent probes were used to label Fe^2+^ in cytoplasm and mitochondria, respectively. After twice washing by phosphate-buffered saline (PBS), the cells were incubated with FerroOrange or Mito-FerroGreen working solution for 30 min at a 37℃ incubator with 5% CO_2_. Lastly, the fluorescent images were captured and analyzed using a laser scanning confocal microscope (LSCM; Nikon, Japan) and Image J software.

### Lipid peroxides measurement

C11-BODIPY^581/591^ (10 μM; Invitrogen, USA) was applied to indicate cellular lipid ROS. The fluorescence images were captured using LSCM (Nikon, Japan). The fluorescence intensity of each image was analyzed using Image J software, and the oxidized/reduced ratio was calculated. And also, the oxidized/reduced fluorescent intensity was quantified using FCM (Becton-Dickinson, USA). Additionally, intracellular MDA and GSH contents were detected following the corresponding commercial kits (Jiancheng, China).

### Immunofluorescent analysis of GPX4

After administering with 50 µg/mL SiNPs, cells were fixed under 4% paraformaldehyde for more than 4 h, incubated with 1% Triton X-100 (Solarbio, China) for transparency at 4℃, and blocked with 10% FBS. Afterward, the cells were incubated with GPX4 primary antibody (Novus, USA) overnight at 4℃, labeled by the corresponding fluorescent rabbit secondary antibody for 1 h in the dark, followed by the nuclei staining by DAPI. Lastly, the fluorescent images were captured by LSCM.

### Quantitative RT-PCR

Total RNA of the rat heart tissues or AC16 cells were extracted using Trizol reagent (Thermo, USA), determined through a microplate reader (BioTek, USA), and then reversely transcribed to cDNA by PrimeScript™ RT reagent kit (TaKaRa, Japan) for mRNA amplification, and by Mir-X™ miRNA First-Strand Synthesis (TaKaRa, Japan) for microRNA amplification following the product manual. Quantitative RT-PCR was accomplished under a real-time PCR machine (Bio-Rad, USA) by using either PowerUp™ SYBR™ Green Master Mix (Thermo, USA) or Mir-X miRNA qRT-PCR TB Green Kit (TaKaRa, Japan). The relative amount of gene expression was quantified with β-actin as the internal reference, whilst the miR-125b-2-3p level was normalized to U6. Primer details are listed in Table S3.

### Western blot assay

The total proteins from rat heart tissues or AC16 cells were extracted by using the Protein Rapid Extraction kit (KeyGEN, China). After protein quantification by the BCA method (Beyotime, China), 30 μg for each sample was used to perform the Western blot assay as described previously. In this study, the primary antibodies for ferritin (heavy chain, FTH1; light chain, FTL), TFRC/TfR, DMT1, ACSL4, GPX4, and SLC7A11 were all purchased from Novus, USA, whilst that for HO-1 and glyceraldehyde-3-phosphate dehydrogenase (GAPDH) from CST, USA. Finally, the imaged blots were quantified by dividing the signal of the tested protein by corresponding GAPDH (the loading control) and normalized to the controls.

### Dual luciferase assay

Dual-Luciferase Reporter Assay System (Promega, USA) was performed to determine the targeting relationship between miR-125b-2-3p and HO-1. HO-1-3’ UTR reporter plasmids (both wild and mutant types) were transfected into cells with the miR-125b-2-3p mimics or negative control (miR-NC), respectively. Thereafter, the luciferase activity was detected, and the fluorescence value was read for data analysis.

### Statistical analysis

All data were expressed as mean ± SD. One-way analysis of variance (ANOVA) followed by Dunnett (two-tailed) or Dunnett’s T3 test, and two-way ANOVA followed by Tukey’s post hoc test were used for statistical significance analysis. A Pearson Correlation analysis (two-tailed) was also carried out, and heatmaps were drawn using R 3.6.1. *p* < 0.05 was defined to be statistically significant.

### Supplementary Information

**Supplementary Material 1.**

## Data Availability

The datasets used and/or analyzed during the current study are available from the corresponding author upon reasonable request.
