# Supplementary material for: Ferroptosis contributing to cardiomyocyte injury induced by silica nanoparticles via miR-125b-2-3p/HO-1 signaling
Source: Part Fibre Toxicol. 2024 Apr 1;21:17. doi: 10.1186/s12989-024-00579-5 (PMC10983742; doi:10.1186/s12989-024-00579-5)
Supplement: Supplementary file 1 — Supplementary Material 1. [file 12989_2024_579_MOESM1_ESM.docx]

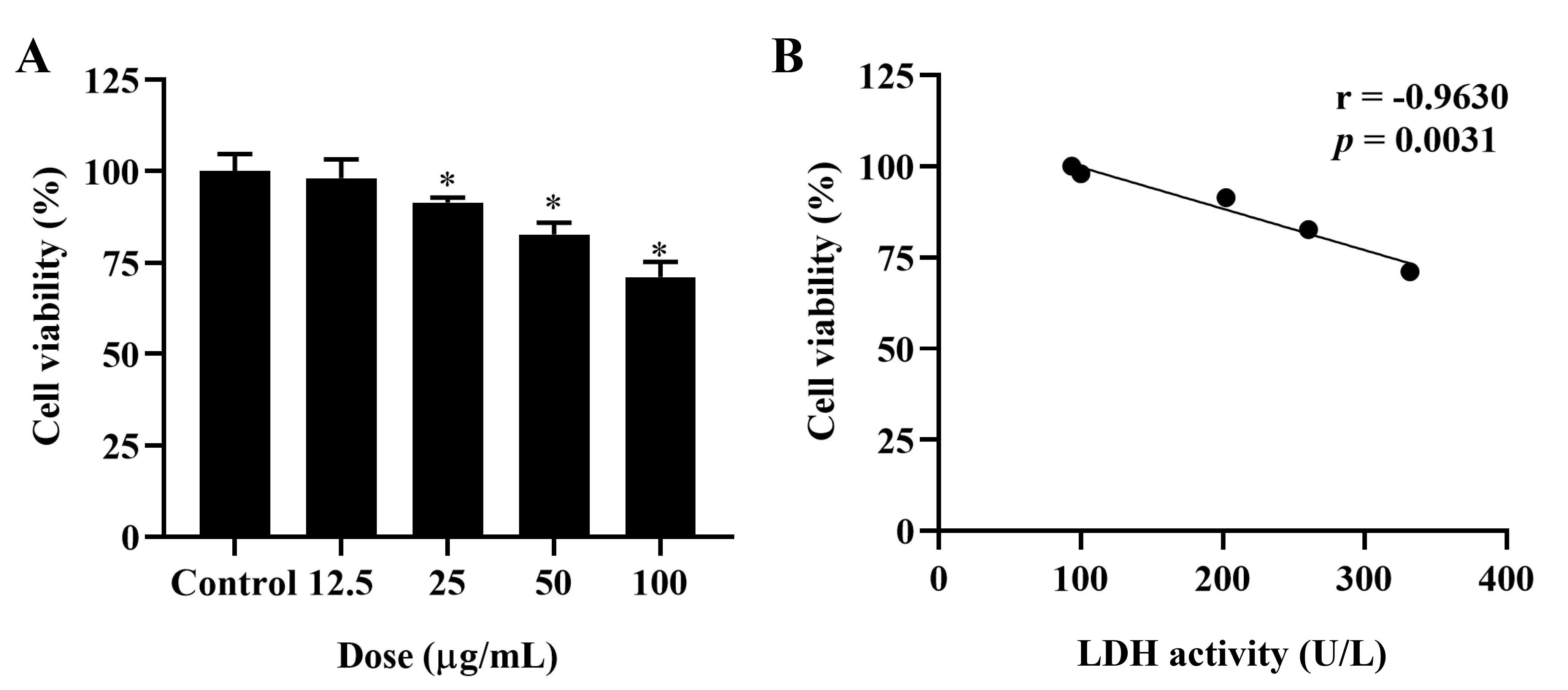


**Fig. S1** SiNPs induced cytotoxicity in AC16 cells. (A) Cell viability. (B) Correlation analysis between cell viability and LDH content. Data were expressed as mean ± SD. ^*^*p* < 0.05 *vs* control.


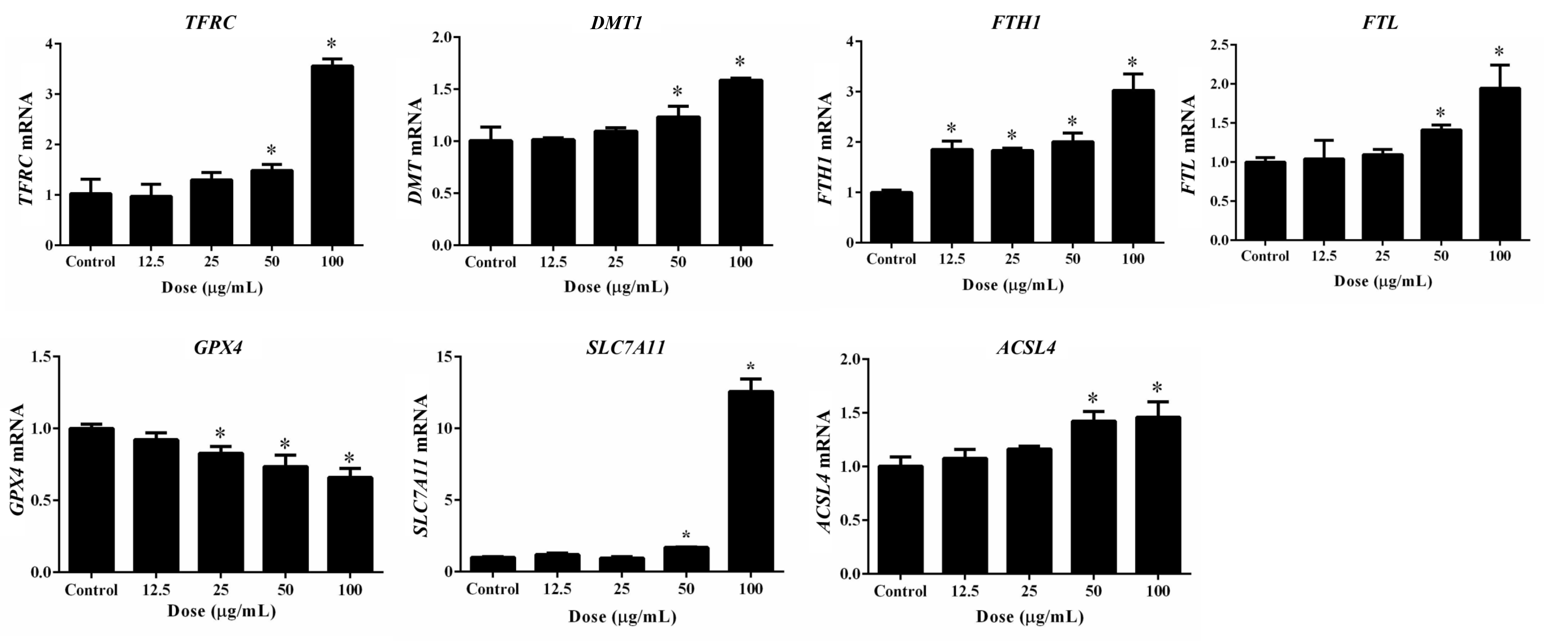


**Fig. S2** SiNPs disturbed the transcriptional expressions of ferroptosis-related genes in AC16 cells. Data were expressed as mean ± SD. ^*^*p* < 0.05 *vs* control.


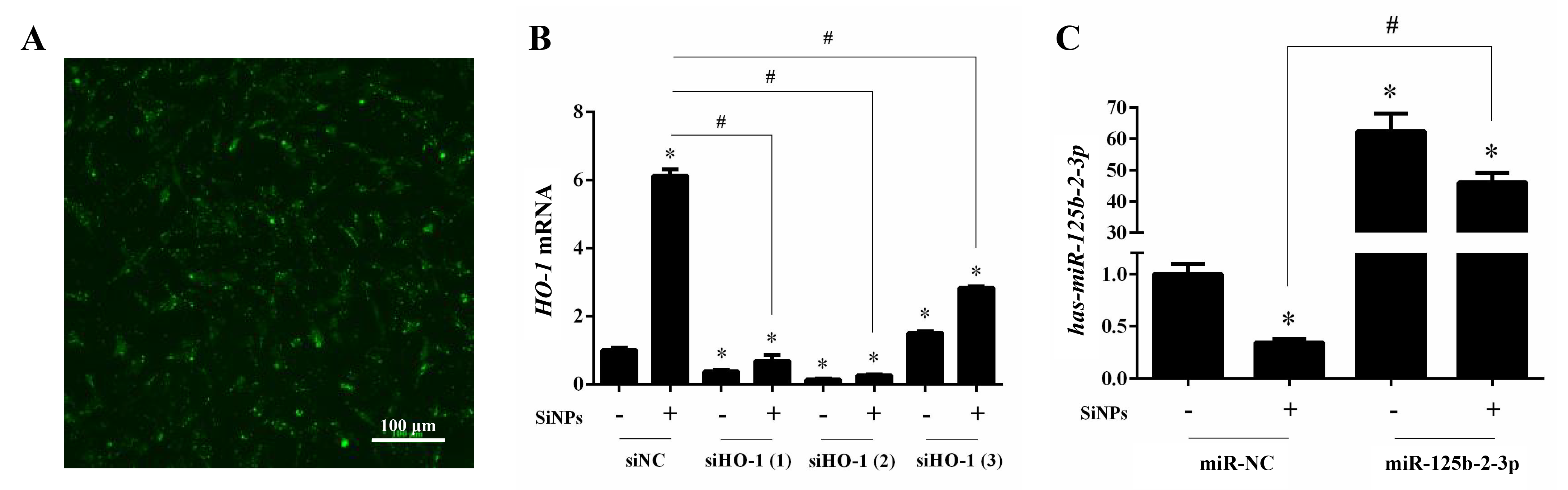


**Fig. S3** The verification of transfection efficacy for siHO-1 or miR-125b-2-3p mimics in AC16 cells. (A) Fluorescent image observation after NC transfection. Scale bar, 100 μm. (B) siHO-1 inhibited mRNA expression of HO-1. (C) miR-125b-2-3p mimics effectively reversed the declined miR-125b-2-3p by SiNPs in AC16 cells. Data were expressed as mean ± SD. ^*^*p* < 0.05 *vs* control, ^#^*p* < 0.05 *vs* SiNPs.


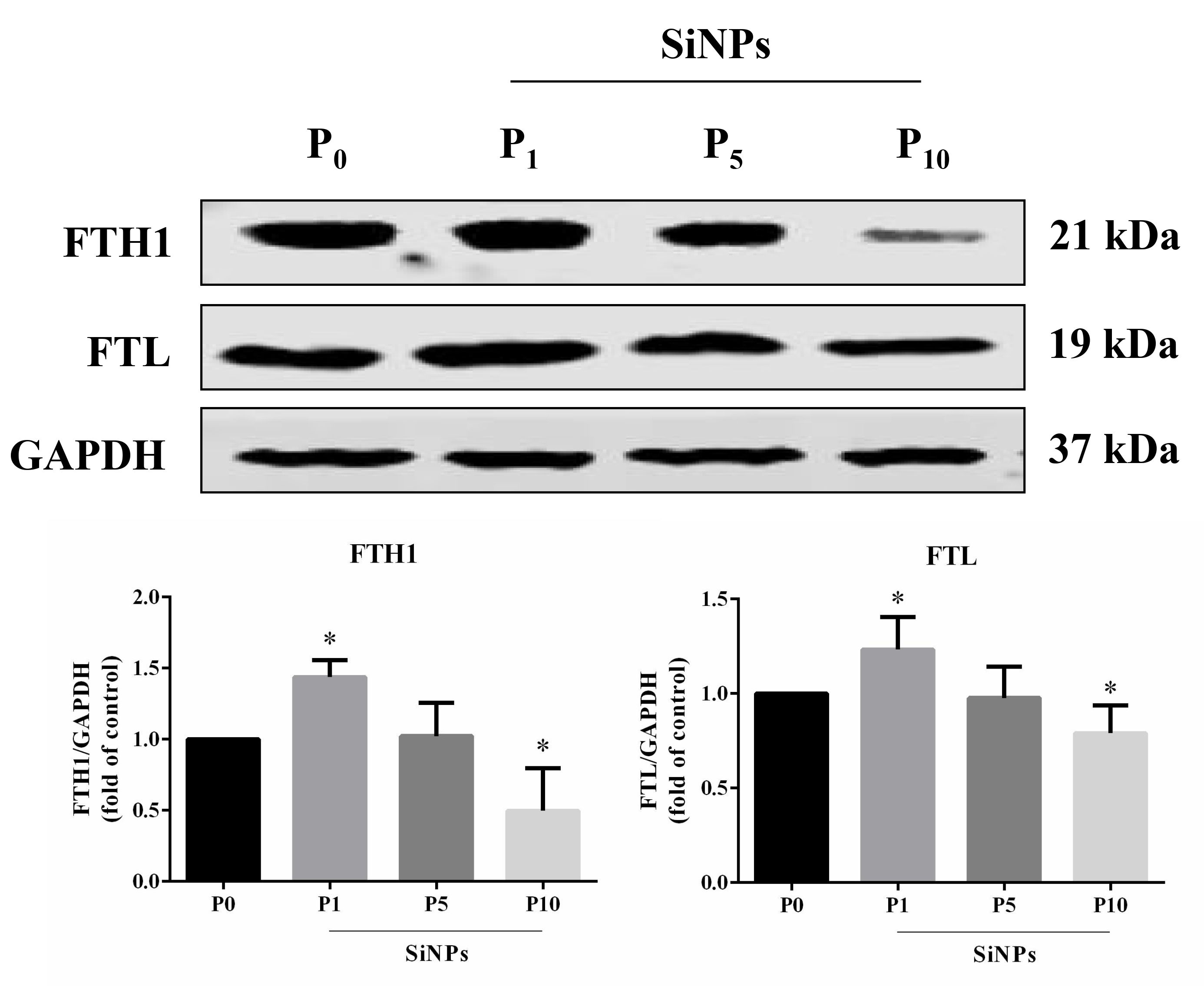


**Fig. S4** The alterations of ferritin expression after the prolonged exposure to SiNPs in AC16 cells. Cells were treated with 50 μg/mL SiNPs up to 10 passages. The expressions of FTH1 and FTL were firstly increased and then decreased with the extension of SiNPs exposure time. Data were expressed as mean ± SD. ^*^*p* < 0.05 *vs* P_0_.


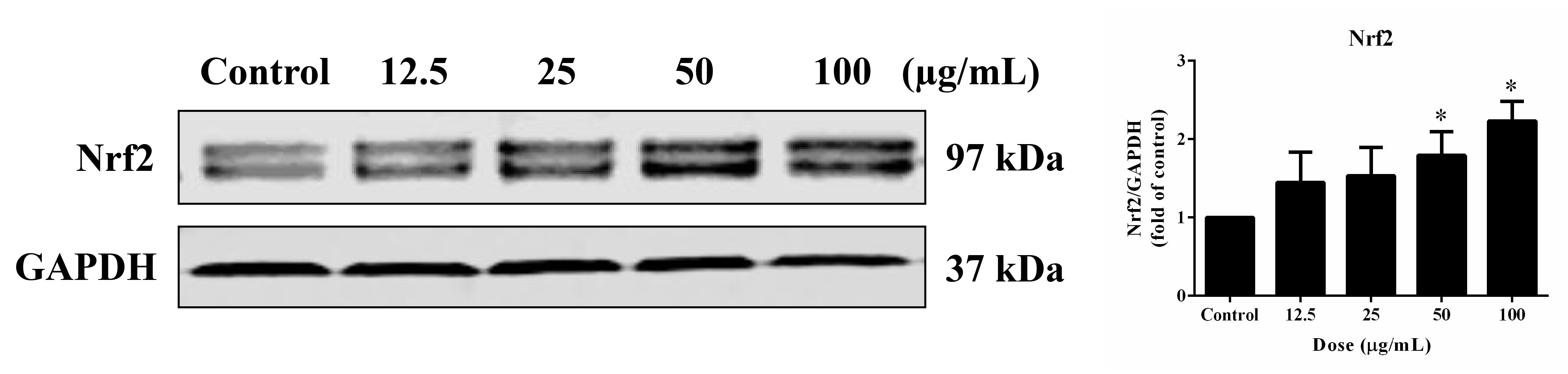


**Fig. S5** The Nrf2 expression was increased induced by SiNPs in AC16 cells. Data were expressed as mean ± SD. ^*^*p* < 0.05 *vs* control, ^#^*p* < 0.05 *vs* SiNPs.


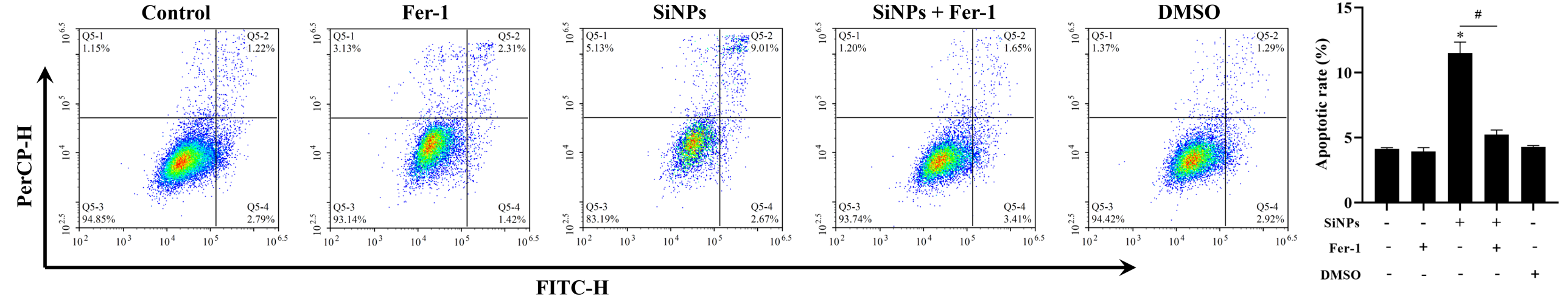


**Fig. S6** Fer-1 alleviated the cell apoptosis induced by SiNPs in AC16 cells. Data were expressed as mean ± SD. ^*^*p* < 0.05 *vs* control, ^#^*p* < 0.05 *vs* SiNPs.

**Table S1** The hydrodynamic sizes and Zeta potentials of SiNPs in different media.

| Dispersion medium | Hydrodynamic size (nm) | Zeta potential (mV) |
| --- | --- | --- |
| Physiological saline | 131.17 ± 2.67 | -31.53 ± 5.12 |
| DMEM/F-12 | 88.50 ± 7.51 | -35.80 ± 0.57 |

Data are expressed as mean ± SD. n = 3.

**Table S2** Transfection sequences

| Gene name | Description | Sequence |
| --- | --- | --- |
| *siNC* | Sense | UUCUCCGAACGUGUCACGU |
|  | Antisense | ACGUGACACGUUCGGAGAA |
| *siHO-1(1)* | Sense | GGCAAUGGCCUAAACUUCA |
|  | Antisense | UGAAGUUUAGGCCAUUGCC |
| *siHO-1(2)* | Sense | CCAAGUUCAAGCAGCUCUA |
|  | Antisense | UAGAGCUGCUUGAACUUGG |
| *siHO-1(3)* | Sense | GGCCAGCAACAAAGUGCAA |
|  | Antisense | UUGCACYYYGUUGCUGGCC |
| *miR-NC* | Sense | UUGUACUACACAAAAGUACUG |
|  | Antisense | GUACUUUUGUGUAGUACAAUU |
| *hsa-miR-125b-2-3p mimics* | Sense | UCACAAGUCAGGCUCUUGGGAC |
|  | Antisense | CCCAAGAGCCUGACUUGUGAUU |

**Table S3** PCR primer pairs

| Gene name | Species | Description | Primer sequence |
| --- | --- | --- | --- |
| *TFRC* | *Homo sapiens* | F | GGCTACTTGGGCTATTGTAAAGG |
|  |  | R | CAGTTTCTCCGACAACTTTCTCT |
| *DMT1* | *Homo sapiens* | F | CATCCTCACATTTACGAGCTTG |
|  |  | R | CCAACCCAAGTAGAACACAAAG |
| *FTH1* | *Homo sapiens* | F | AGAACTACCACCAGGACTCAGAGG |
|  |  | R | AGCCACATCATCGCGGTCAAAG |
| *FTL* | *Homo sapiens* | F | ACCGCGATGATGTGGCTCTG |
|  |  | R | GCTTTCATGGCGTCTGGGGT |
| *GPX4* | *Homo sapiens* | F | GAGGCAAGACCGAAGTAAACTAC |
|  |  | R | CCGAACTGGTTACACGGGAA |
| *ACSL4* | *Homo sapiens* | F | ACTGGCCGACCTAAGGGAG |
|  |  | R | GCCAAAGGCAAGTAGCCAATA |
| *SLC7A11* | *Homo sapiens* | F | TGCCTTCCCTGGGCAACAAG |
|  |  | R | GTGTTCTGGAGCACGCCCTT |
| *HO-1* | *Homo sapiens* | F | CTTCTTCACCTTCCCCAACAT |
|  |  | R | TTCTATCACCCTCTGCCTGACT |
| *Nrf2* | *Homo sapiens* | F | ATTGCCTGTAAGTCCTGGTCAT |
|  |  | R | GCTTTTGCCCTAAGTTCATCTC |
| *β-actin* | *Homo sapiens* | F | GAGACCTTCAACACCCCAGC |
|  |  | R | ATGTCACGCACGATTTCCC |
| *rno-miR-125b-2-3p* | *Rattus norvegicus* | F | AGCCAGCGACAAGTCAGGCTCT |
|  |  | R | TakaraBio |
| *hsa-miR-125b-2-3p* | *Homo sapiens* | F | CTCACAAGTCAGGCTCTTGGGAC |
|  |  | R | TakaraBio |
| *U6* |  | F | GGAACGATACAGAGATTAGC |
|  |  | R | TGGAACGCTTCACGAATTTGCG |
